# Conversion of unresponsiveness to immune checkpoint inhibition by fecal microbiota transplantation in patients with metastatic melanoma: study protocol for a randomized phase Ib/IIa trial

**DOI:** 10.1186/s12885-022-10457-y

**Published:** 2022-12-30

**Authors:** J. S. W. Borgers, F. H. Burgers, E. M. Terveer, M. E. van Leerdam, C. M. Korse, R. Kessels, C. C. Flohil, C. U. Blank, T. N. Schumacher, M. van Dijk, J. G. E. Henderickx, J. J. Keller, H. W. Verspaget, E. J. Kuijper, J. B. A. G. Haanen

**Affiliations:** 1 Department of Medical Oncology, Antoni Van Leeuwenhoek, The Netherlands Cancer Institute, Plesmanlaan 121, 1066 CX Amsterdam, The Netherlands; 2 Netherlands Donor Feces Bank, Department of Medical Microbiology, Leiden University Medical Center, Leiden, The Netherlands; 3 Center for Microbiome Analyses and Therapeutics at Department of Medical Microbiology, Leiden University Medical Center, Leiden, The Netherlands; 4 Department of Gastrointestinal Oncology, Antoni Van Leeuwenhoek, The Netherlands Cancer Institute, Amsterdam, The Netherlands; 5 Department of Gastroenterology and Hepatology, Leiden University Medical Center, Leiden, The Netherlands; 6 Department of Laboratory Medicine, Antoni Van Leeuwenhoek, The Netherlands Cancer Institute, Amsterdam, The Netherlands; 7 Department of Biometrics, Antoni Van Leeuwenhoek, The Netherlands Cancer Institute, Amsterdam, The Netherlands; 8 Department of Pathology, Antoni Van Leeuwenhoek, The Netherlands Cancer Institute, Amsterdam, The Netherlands; 9 Division of Molecular Oncology and Immunology, Oncode Institute, The Netherlands Cancer Institute, Amsterdam, The Netherlands; 10 Department of Hematology, Leiden University Medical Center, Leiden, The Netherlands; 11 Clinical Trial Service Unit, Antoni Van Leeuwenhoek, The Netherlands Cancer Institute, Amsterdam, The Netherlands; 12 Department of Gastroenterology, Haaglanden Medical Center, Den Haag, The Netherlands; 13 Department of Biobanking, Leiden University Medical Center, Leiden, The Netherlands

**Keywords:** FMT, Gut microbiome, Immunotherapy, Anti-PD-1, Melanoma

## Abstract

**Background:**

The gut microbiome plays an important role in immune modulation. Specifically, presence or absence of certain gut bacterial taxa has been associated with better antitumor immune responses. Furthermore, in trials using fecal microbiota transplantation (FMT) to treat melanoma patients unresponsive to immune checkpoint inhibitors (ICI), complete responses (CR), partial responses (PR), and durable stable disease (SD) have been observed. However, the underlying mechanism determining which patients will or will not respond and what the optimal FMT composition is, has not been fully elucidated, and a discrepancy in microbial taxa associated with clinical response has been observed between studies. Furthermore, it is unknown whether a change in the microbiome itself, irrespective of its origin, or FMT from ICI responding donors, is required for reversion of ICI-unresponsiveness. To address this, we will transfer microbiota of either ICI responder or nonresponder metastatic melanoma patients via FMT.

**Methods:**

In this randomized, double-blinded phase Ib/IIa trial, 24 anti-PD1-refractory patients with advanced stage cutaneous melanoma will receive an FMT from either an ICI responding or nonresponding donor, while continuing anti-PD-1 treatment. Donors will be selected from patients with metastatic melanoma treated with anti-PD-1 therapy. Two patients with a good response (≥ 30% decrease according to RECIST 1.1 within the past 24 months) and two patients with progression (≥ 20% increase according to RECIST 1.1 within the past 3 months) will be selected as ICI responding or nonresponding donors, respectively. The primary endpoint is clinical benefit (SD, PR or CR) at 12 weeks, confirmed on a CT scan at 16 weeks. The secondary endpoint is safety, defined as the occurrence of grade ≥ 3 toxicity. Exploratory endpoints are progression-free survival and changes in the gut microbiome, metabolome, and immune cells.

**Discussion:**

Transplanting fecal microbiota to restore the patients’ perturbed microbiome has proven successful in several indications. However, less is known about the potential role of FMT to improve antitumor immune response. In this trial, we aim to investigate whether administration of FMT can reverse resistance to anti-PD-1 treatment in patients with advanced stage melanoma, and whether the ICI-responsiveness of the feces donor is associated with its effectiveness.

**Trial registration:**

ClinicalTrials.gov: NCT05251389 (registered 22-Feb-2022). Protocol V4.0 (08–02-2022).

**Supplementary Information:**

The online version contains supplementary material available at 10.1186/s12885-022-10457-y.

## Background

### Introduction

For patients with advanced stage melanoma, treatment possibilities have increased considerably in the past decade. Since the introduction of immune checkpoint inhibitors (ICI) blocking the inhibitory T cell checkpoints PD-1 and CTLA-4, either sequentially or combined, five-year overall survival (OS) rates of up to 50% have been observed in patients with advanced stage melanoma [1]. More recently, a novel combination of anti-LAG-3 plus anti-PD-1 led to improved progression-free survival compared to anti-PD-1 alone [2]. Despite these impressive results, the majority of patients with metastatic melanoma still succumbs to the disease. Ample research of the tumor microenvironment (TME) has revealed several escape mechanisms. However, novel drugs or combinations of drugs to overcome these resistance mechanisms have not yet been approved and major breakthroughs are still lacking. Therefore, other mechanisms to improve the antitumor immune responses are currently being explored. One such a mechanism may be the manipulation of the microbiome of immunotherapy-resistant patients.

### Preclinical and observational data

The first evidence that the gut commensal microbial composition plays an important role in immune responses, including antitumor immunity and responses to ICI, came from preclinical studies. Investigators noticed that C57BL/6 mice derived from different vendors, with known differences in their commensal microbiota, showed differences in spontaneous antitumor immunity upon inoculation of B16 melanoma tumor cells [3]. Fecal microbiota transplantation (FMT) from mice with an increased relative abundance of commensal *Bifidobacterium* spp. (with a similarity of 99% *to B. breve, B. longum*, and B*. adolescenti*) to the other group was associated with delayed tumor growth, to a similar degree as observed upon anti-PD-1 treatment. Combined treatment showed superior activity in these mice [3].

Using 16S ribosomal ribonucleic acid (rRNA) gene amplicon sequencing and shotgun metagenomic sequencing, multiple attempts have been made to identify similar gut microbiome characteristics associated with response to ICI treatment in patients [4–10]. Thus far, several bacterial species, including *Akkermansia muciniphila, Bifidobacterium bifidum* and *adolescentis, Barnesiella intestinihominis, Alistipes* species (spp.), *Faecalibacterium* and other Firmicutes, such as *Ruminococcus* spp. and *Blautia* spp., have been associated with response to ICI [4–11]. However, as certain taxa, including *Akkermansia muciniphila*, *Ruminococcus* spp and *Bacteroides* spp., have also been associated with nonresponse [7, 8], it has been proven difficult to pinpoint a specific (combination of) taxa to either response or nonresponse. Notably all studies focussed on bacterial gut microbiota and did not perform analyses of Archeae, fungi or viruses.

A second hypothesis has been that (non)response is not determined by specific taxa, but rather by the diversity and functional differences of the gut microbiome. This is supported by data indicating that patients with a high microbiome diversity have a longer progression-free survival (PFS), as compared to patients with low or intermediate diversities [5, 12]. Furthermore, it has been shown in both mice and patients that the use of antibiotics prior to or shortly after initiation of ICI treatment is associated with worse outcomes compared to control groups that did not receive antibiotic treatment [11, 13–19]. Similar findings have been observed for patients using proton-pump inhibitors (PPI) prior to start of ICI treatment [19]. These data suggest that also antibiotic- or PPI-associated microbiota changes of the gut microbiota can compromise the efficacy of ICI [6, 11, 13–19].

Studies in melanoma patients have used metagenomic functional pathway analysis and metabolomics to gain functional insights into gut microbiome profiles associated with efficacy of ICI [12, 20, 21]. Various functional pathways of the microbiome have been associated with PFS, including protective pathways of amino acid biosynthesis, as well as risk-associated pathways of sugar degradation, guanosine nucleotide biosynthesis and B vitamin biosynthesis [12, 21, 22]. Although metagenomics provides insight into the functional potential of the microbiome, metabolomics allows to identify metabolites released by the microbial community. Thus far, metabolomic profiling has not been performed as extensively as metagenomic functional pathway analysis and solely identified high levels of the plant xenobiotic anacardic acid in ICI responders [20]. Future studies could use a complementary -omics approach to elucidate the functional potential and active processes of the gut microbiome with respect to ICI responsiveness.

To further investigate the impact of the gut microbiome on antitumor responses, several studies have transplanted feces from ICI responding (R) and nonresponding (NR) patients to mice prior to tumor cell inoculation [4, 5, 11, 23]. In the majority of mice, treatment with R-FMT led to reduced tumor sizes, an effect that could be enhanced by additional treatment with anti-PD-(L)1 [4, 5, 11, 23]. Response correlated with donor-recipient microbiota similarity (“FMT take”), higher density of CD8+ T cell infiltration, increased number of CD8+ T cells in the gut, higher systemic levels of effector CD4+ and CD8+ T cells, and lower levels of regulatory T cells (Tregs) and myeloid-derived suppressor cells (MDSC) [4, 5, 11].

### Clinical trials

The convincing preclinical and observational data, together with the previously observed successes of FMT in the treatment of Clostridioides difficile infection, ulcerative colitis and graft-versus-host disease, formed the basis of the first FMT clinical trials in the field of oncology [24–26]. Recent published data of two phase I trials show that FMT and reintroduction of anti-PD-1 treatment in refractory metastatic melanoma patients is safe, feasible, and can be effective [27, 28]. The patients received an FMT via colonoscopy, with or without pretreatment with broad-spectrum antibiotics (to enhance engraftment) and maintenance cycles of oral stool capsules. Out of the 26 patients, 6 had an objective response (complete response (CR), partial response (PR)), and an additional 3 patients had clinical benefit (stable disease (SD) > 12 months), which was associated with longer PFS and OS [27, 28].

Interestingly, in the study from Baruch et al*.*, using two metastatic melanoma patients with a CR as FMT-donors, only patients treated with FMTs from donor 1 showed objective responses [27]. Both donors had previously reportable immunotherapy-favorable features, but donor 1 had a lower microbiota diversity than donor 2. 16S rRNA gene sequencing and metagenomics revealed that patients who received an FMT from donor 1 had a higher relative abundance of *Bifidobacterium adolescentis*, whereas *Ruminococcus bromii* was more abundant in patients receiving an FMT from donor 2. Post FMT, the gut microbiota of responders were characterized by a higher relative abundance of *Enterococcaceae*, and *Streptococcus australis,* and a lower relative abundance of *Veillonella atypica*. However, as similar taxa were found in some nonresponders, the authors were unable to reveal a clear association between taxa and clinical response. Important to note is that this study was statistically powered to assess safety, and was therefore not designed to compare efficacy between donors [27].

The study by Davar et al*.* used both CR and PR patients as FMT donors and did not observe any differences in CR/PR donor stool [28]. Shotgun metagenomic sequencing did reveal that in all responders, the microbiota composition shifted significantly towards the composition of the donor, while this was only observed in about half of the nonresponders. Single-cell analyses showed that responders had higher percentages of CD56^+^CD8^+^ T cells (subset of activated CD8+ T cells with higher cytolytic functions [29]) on day 42 post-FMT, while nonresponders showed higher frequencies of myeloid cells and Tregs [28].

Next to using FMT as a way of modulating the gut microbiome to enhance response to ICI, Dizman et al*.* performed a randomized trial for patients with metastatic renal cell carcinoma in which they looked at the effects of ipilimumab-nivolumab with or without CBM588, a live bacterial supplementation containing *Clostridium butyricum* [10]. In this trial, the addition of CBM588 containing a bacterial strain producing butyrate, led to higher objective response rates and prolonged PFS. Further analysis of the gut microbiota of responding patients revealed an increase in *Bifidobacterium* spp., which has previously been associated with increased anti-tumor immunity in preclinical studies [3].

### Summary

In conclusion, manipulation of the gut microbiome and metabolome by donor fecal microbiota transplantation may influence the antitumor immune response. However, the exact mechanisms linking commensal bacteria, fungi or bacteriophages to the anticancer efficacy of immune checkpoint blockade are unresolved [30]. Thus far, discrepancies have been found between studies, in which certain bacterial taxa that are considered favorable in one study, are associated with lack of response in an independent study [4–11]. Similarly, the optimal FMT composition remains to be elucidated. It is assumed that fecal microbiota transplantation from ICI responding patients may enhance the capacity of ICI nonresponders to respond to subsequent ICI treatment. Likewise, it is thought that transplantation of feces from ICI nonresponding patients may prevent a response to ICI treatment. However, it is currently unknown whether the background of the FMT donor matters. In order to address the question whether simply a change in microbiome, irrespective of its origin, or an FMT from an ICI-responding donor patient is required for reversion of ICI unresponsiveness, we will perform a randomized controlled phase Ib/IIa study. In this trial, we will transfer fecal microbiota originating from either responding or nonresponding metastatic melanoma patients via FMT while continuing with immunotherapy (anti-PD-1), with the aim to revert ICI unresponsiveness of anti-PD-1 refractory advanced stage melanoma patients.

## Methods/design

**Fig. 1:**
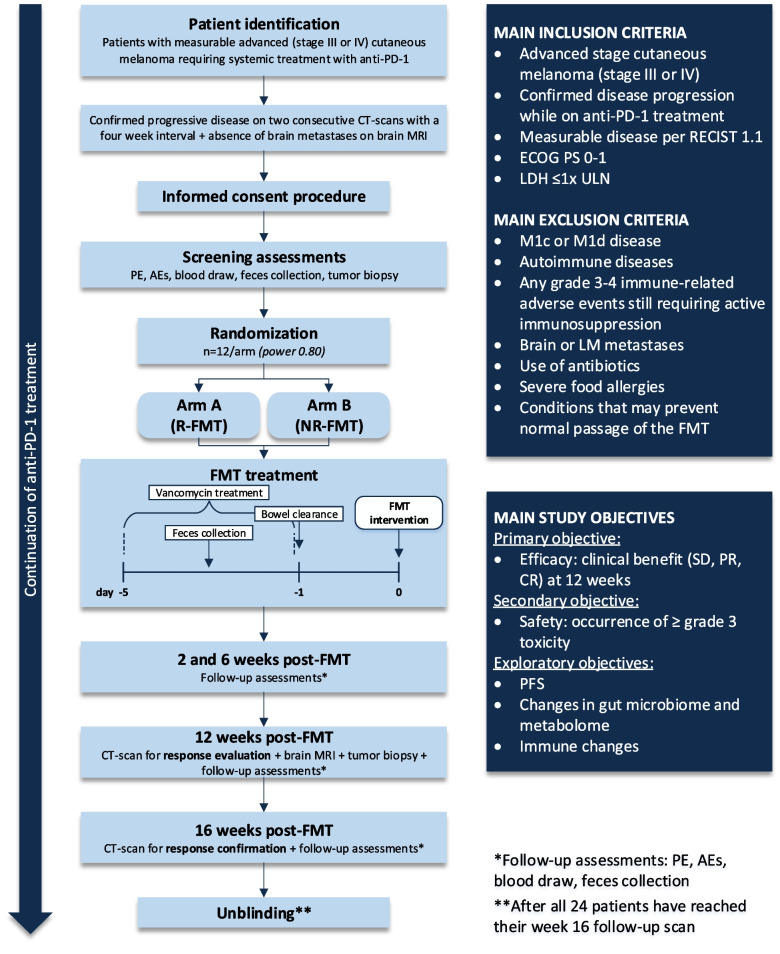
Flowchart

This is a single-center, randomized, double-blinded phase Ib/IIa trial investigating FMT from ICI responding or ICI nonresponding donors to patients with anti-PD-1 refractory advanced stage melanoma. In total, four metastatic melanoma patients will be selected as donors: two anti-PD-1 responders and two anti-PD-1 nonresponders. Up to 24 anti-PD-1 refractory melanoma patients will be enrolled to randomly receive an FMT from either a responding or nonresponding donor with continuation of their anti-PD-1 treatment (Fig. 1). Patients will be enrolled and treated at the Netherlands Cancer Institute (NKI, Amsterdam, the Netherlands). Donor feces will be processed into fecal suspensions for FMT at the Netherlands Donor Feces Bank (NDFB, Leiden University Medical Center (LUMC), the Netherlands). The total duration of the study will be approximately two years.

### Study design

#### Endpoints

##### Primary endpoint

- Efficacy, defined as clinical benefit (SD, PR, CR) at 12 weeks, confirmed on a second CT scan at 16 weeks, of an FMT-intervention from ICI responding or nonresponding donors in anti-PD-1 refractory advanced stage melanoma patients.

##### Secondary endpoint

- Safety, defined as the occurrence of toxicity grade 3 or higher. The study will be considered safe if less than seven patients have experienced grade ≥ 3 toxicity.

##### Exploratory endpoints

- Progression-free survival (PFS, according to RECIST 1.1), calculated from the date of registration to date of progression or death, whichever occurs first, censoring patients without progression and who are still alive at last follow-up.
- Changes in the gut microbiome and metabolome following FMT, and the duration and stability over time.
- Immune changes, including changes in cell populations (absolute, relative, phenotypical), in chemokine/cytokine levels and in the TME.

#### Selection and screening of donors

A total of four patients with metastatic, cutaneous melanoma treated with anti-PD-1 therapy will be selected as donors. Two patients with an ongoing complete or partial remission on anti-PD-1 treatment, defined as ≥ 30% decrease or disappearance of all lesions according to RECIST 1.1 within the past 24 months, will be selected as ICI responding donors (group R). From patients who developed a confirmed progressive disease as best response, defined as ≥ 20% increase according to RECIST 1.1 within the past three months, two additional donors will be selected as ICI nonresponding donors (group NR). Patients cannot be selected as donors if they are known to have an auto-immune disease or metabolic syndrome, have recently used antibiotics (within three months prior to feces donation) or have experienced an immune-related adverse event (irAE) requiring systemic treatment. Donors will be screened for active blood- or feco-transmissive diseases and recent risk behavior as described before by the NDFB [31]. These screening guidelines are in line with the European consensus guideline [32]. Three to five weeks after the fecal donations used for FMT preparations, donors will be rescreened. After passing the 2^nd^ screening round, stool suspensions are released for clinical use.

#### FMT manufacturing

Donors will collect feces for donation using a FECOTAINER® (AT Medical BV, The Netherlands) to prevent environmental contamination. The donor feces will be processed into FMT preparations at the laboratory of the NDFB as described previously [33]. In brief, within six hours of defecation, the NDFB will process the collected feces into ready-to-use fecal suspensions, with physiologic saline by homogenization and filtration using a stomacher with filterbag. After addition of glycerol in an endvolume of 10%, the fecal suspensions are stored at -80 °C in the centralized LUMC biobank facility. On the day prior to the FMT, the fecal suspensions will be thawed overnight at 4˚C. 30 to 60 min prior to the FMT administration, the suspensions are transferred into 50 mL syringes and kept at room temperature until administration. Thawed suspensions have to be infused on the same day and cannot be refrozen.

#### Patient selection and eligibility

In total, 24 patients with advanced stage cutaneous melanoma with disease progression, defined as ≥ 20% increase or measurable recurrence according to RECIST 1.1, while on anti-PD-1 monotherapy treatment (adjuvant or first line palliative) will be included in this study. Prior to enrollment, disease progression has to be confirmed on a second scan, four weeks after the initial scan showing progression. Main exclusion criteria are the presence of brain metastases or leptomeningeal (LM) disease, an elevated LDH level (defined as > 1 × ULN), use of antibiotics within three months prior to FMT or expected use of antibiotics during the study, history of autoimmune disease, severe food allergies or conditions that may prevent normal passage of the FMT. All in- and exclusion criteria are listed in Table 1.

**Table 1:** Inclusion and exclusion criteria for participating patients

**Inclusion criteria**
- Age of 18 years or older - Pathologically confirmed advanced stage cutaneous melanoma (stage III or IV) requiring systemic treatment with anti-PD-1: ○ In case of stage IV disease, only patients with M1a or M1b disease are eligible - Confirmed disease progression (≥ 20% increase or measurable recurrence according to RECIST 1.1) on two consecutive scans with a four-week interval while on anti-PD-1 treatment, of which the second scan has to be performed within three weeks prior to signing informed consent - Measurable disease per RECIST 1.1 criteria - ECOG performance status of 0–1 - Life expectancy of > three months - Adequate organ function as determined by standard-of-care lab (including serum ALT/AST < 3 × the upper limit of normal (ULN); serum creatinine clearance ≥ 50 mL/min; total bilirubin ≤ 20 μmol/L, except in patients with Gilbert’s Syndrome who must have a total bilirubin < 50 μmol/L) - LDH level of ≤ 1 × ULN - Use of highly effective method of birth control during treatment (for both genders) - Able to understand and sign the informed consent form
**Exclusion criteria**
- Acral, uveal or mucosal melanoma or an unknown primary - Previous treatment for melanoma other than anti-PD-1 treatment - Stage IV with M1c or M1d disease - Autoimmune diseases (e.g. history of inflammatory bowel disease, including ulcerative colitis and Crohn’s disease (this does not include Hashimoto thyroiditis, vitiligo, or history of psoriasis without active disease)) - Any grade 3 or 4 irAE still requiring active immunosuppressive medication, apart from endocrinopathies that are stable under hormone replacement therapy. Patients who have developed grade 3–4 irAEs, which have reverted to grade 1 with immunosuppressive drugs and who are off immunosuppression at least two weeks prior to enrollment are eligible - Brain or LM metastasis - Elevated LDH level - History of major gastric, esophageal or bowel surgery (e.g. Wipple procedure, subtotal colectomy) - Severe food allergy (e.g. nuts, shellfish) - Swallowing disorders or expected bowel passage problems (e.g. ileus, fistulas, perforation) - Severe dysphagia with incapability of swallowing one liter of bowel lavage - Life expectancy of < three months - Severe cardiac or pulmonary comorbidities (per judgement of the investigator) - Women who are pregnant or breastfeeding - Active systemic infections, coagulation disorders or other active major medical illnesses - Other malignancies, except adequately treated and with a cancer-related life expectancy of > five years - Treatment with antibiotics in the three months prior to study enrollment, or expectation to receive antibiotics during the course of this study

#### Registration, randomization and treatment allocation

After providing informed consent, potential donors and patients will undergo the prespecified screening procedures (Fig. 1). Eligibility must be verified by authorized staff members of the Netherlands Cancer Institute Trial Office prior to registration. Upon registration, both donors and patients will be assigned a unique study-ID number. In total 24 FMT suspensions will be prepared from four donors, meaning a total of six suspensions per donor. The NDFB will randomize the FMT suspensions using a block randomization technique with a block size of four, including one FMT from each donor per block. After randomization, the fecal suspensions will be labeled with numbers 1–24, corresponding with the 24 study participants receiving the FMTs. Treatment allocation will be based on date of study enrollment and the corresponding study-ID number. There will be no stratification factors.

#### Blinding

This is a double-blinded study, meaning that neither the patients nor the staff involved at the operating center (NKI) will know from which donor the FMT suspensions are derived. Concealed allocation will be guaranteed, as the fecal-suspension numbers (1–24) will be the only information that the personal at the operating center will receive during the course of the study. Only the NDFB will have access to the randomization key. In case of a serious adverse event (SAE), or in case an interim analysis has to be performed, the key will be shared with the Data and Safety Monitoring Board (DSMB).

#### Study assessments

If possible, study assessments will be combined with routine clinic visits. At baseline, pre-FMT, and 2 weeks, 6 weeks, 12 weeks and 16 weeks after FMT treatment patients will be monitored using physical exams and laboratory testing. Data will be recorded in the electronic Case Report Forms (eCRF).

##### Imaging assessments

Within three weeks prior to study enrollment, a CT scan (thorax and abdomen) and a brain MRI will be made to confirm disease progression and exclude the presence of brain metastases, respectively. At 12 weeks after the FMT treatment, patients will undergo a second CT scan and brain MRI for response evaluation. The response will be confirmed on a third CT scan at week 16. Response will be evaluated according to RECIST 1.1 criteria.

##### Safety reporting

Safety will be evaluated by documentation of (S)AEs. Adverse events of grade 2 and higher, both related and unrelated to the FMT and anti-PD-1 treatment, will be captured up until 100 days post-FMT. After 100 days, SAEs will only be reported (until two years post-FMT) if, in the opinion of the investigator, the FMT or a protocol procedure may have caused the event. All documented AEs will be followed until resolved to a grade 1 toxicity, or lower. AEs will be graded according to CTCAE v5.0.

##### Biological sample collection for translational research

To assess changes in the gut microbiome composition and metabolome, feces samples will be collected at baseline, prior to FMT, and at multiple time points after FMT (Fig. 1). Patients collect feces at home up to 24 h before a hospital visit by using a feces collection kit with feces containers (BRAND® 62,350, Germany) and store it at 4˚C. On the day of the hospital visit, the feces will be divided unprocessed in four 1.5 mL tubes (Sarstedt, Germany) for future microbiota analyses. Four other tubes will be filled with feces mixed with glycerol in an end volume of 10% for future culturomics. The fecal aliquots will be stored at -80 °C.

Additionally, questionnaires developed for this study will be collected providing information regarding dietary patterns and the time of defecation (Supplementary file 1). Blood samples will be taken at several time points throughout the study for investigation of systemic immune changes. To analyze local immune changes in the TME, tumor biopsies will be taken at baseline and 12 weeks after FMT treatment (Fig. 1).

#### FMT treatment

After study enrollment, patients receive oral vancomycin 250 mg four times daily for four days (day -5 up until day -2) to facilitate engraftment of the donor microorganisms. On the day prior to the FMT patients undergo bowel clearance by drinking 1L of MoviPrep. On day 0, the FMT will be administered by a gastroenterologist using esophagogastroduodenoscopy. Through the gastroscope a total amount of 198 ml fecal suspension, containing 60 g of processed donor feces, will be transplanted in the duodenum. The suspension will be injected in a partially upright position of the patient to prevent regurgitation. During the entire study period anti-PD-1 treatment will be continued according to the patient’s regular treatment schedule.

#### Translational research

The collected fecal samples will be processed and analyzed to assess changes in the fecal microbiome (including bacteria, archaea and fungi) and metabolome (metabolites, such as amino acids, alcohols, polyols, organic acids, as well as nucleotides and vitamins). Microbial DNA will be extracted from 0.1 g feces using the Quick-DNA™ Fecal/Soil Microbe Miniprep Kit (ZymoResearch, CA, USA). Negative extraction controls will be included, as well as positive extraction and sequencing controls using the ZymoBIOMICS Microbial Community Standard and the ZymoBIOMICS Microbial Community DNA Standard, respectively. Shotgun metagenomics will be performed on the Illumina platform according to the manufacturer’s protocols. Raw sequencing data will be processed using computational pipelines for taxonomic and functional profiling. The obtained data will be linked to clinical characteristics. For assessment of the metabolome, feces samples will be processed and analyzed by Liquid Chromatography Tandem Mass Spectrometry (LC–MS/MS) or Nuclear Magnetic Resonance (NMR). The identified metabolic profile will be correlated to microbiome data and clinical characteristics.

Collected tumor biopsies and blood samples will be used for investigation of the local and systemic immunological changes, respectively. This will involve investigating changes in immune cell populations (absolute, relative, phenotypic), in chemokine/cytokine levels and in the TME. The results will be linked to treatment response.

#### Sample size

The sample size calculation is based on the intention-to-treat analysis of the primary endpoint (clinical benefit at 12 weeks). Based on the response rates in the two previously conducted phase I trials in anti-PD-1 refractory metastatic melanoma patients, we assume that the percentage of patients having clinical benefit at week 12 in the group receiving R-FMT will be 50%, compared to 5% for the group receiving NR-FMT [27, 28]. With a sample size of 12 patients per group, the comparison of the clinical benefit probabilities between the groups will then have 80% power, when using Fisher's exact test with a two-sided significance level of 0.20. If the true percentage of patients having clinical benefit in the NR-FMT group is 1%, the power of the test will be approximately 90%. Due to safety reasons, it was decided to include a limited number of patients. Hence, the unusually low confidence level is considered defensible, as it allows us to still identify a potential difference in effectiveness between two small groups with sufficient power. If the outcome of this study is positive, larger numbers of patients can be included in a potential follow-up study, using the usual confidence levels. If it turns out that there is no difference between the two groups, both arms will also be compared separately against a low response rate of 0.05 using a one-sample proportion test.

#### Statistical analysis methods

Once all 24 patients have reached their week 16 follow-up scan, the treatment arms will be deblinded and compared. Demographics, and patient, microbiome and tumor characteristics will be presented per treatment arm. Differences in continuous outcomes and categorical variables will be tested using the nonparametric Kruskal–Wallis test and the Fisher Exact test, respectively. Outcomes from statistical analyses will be corrected for multiple-testing whenever appropriate.

##### Primary endpoint

The primary endpoint, clinical benefit (SD, PR or CR) at 12 weeks and confirmed on a second scan at 16 weeks, will be analyzed according to the intention-to-treat principle and by using the Fisher Exact test.

##### Secondary endpoint

The secondary endpoint is safety of the FMT-intervention. Safety will be analyzed in all randomized patients according to the actual received treatment (‘as treated’). AEs will be tabulated using descriptive statistics (number/percentage of patients) according to the worst grade toxicity per AE per patient. A distinction will be made between FMT-related AEs and AEs associated with anti-PD-1 treatment.

##### Exploratory endpoints

The exploratory endpoints are the PFS, changes in the gut microbiome and metabolome, and immune changes. PFS curves will be constructed using the Kaplan–Meier method and only reported descriptively, given the small sample size and short study duration. The changes in the gut microbiome and metabolome will be assessed by statistical analyses and data visualization in R using packages phyloseq, vegan, ggplot2, and Microbiome, among others. This methodology will provide insight in microbiota composition and microbiota richness/diversity at various taxonomic levels, which can be linked to clinical variables and outcomes. The results of laboratory investigations (continuous variables) will be expressed as means with their standard deviation.

#### Interim analysis

Anti-PD-1 treatment is known to induce grade 3–4 toxicity in approximately 15% of patients [34]. With a total sample size of 24 patients, the probability of grade 3–4 toxicity in ≥ 7 patients is 5.7% (using the exact binomial distribution). Therefore, if > 6 patients (25%) experience such toxicity this would raise concerns about the safety of the treatment. A DSMB will be installed for this study comprising members without a conflict of interest with the sponsor of the study. The DSMB will meet for final analysis, and in case > 6 patients experience grade 3/4 toxicities. In this latter case, an interim analysis will be performed to investigate the risk–benefit ratio per treatment arm and to decide whether the study can continue.

#### Ethics and dissemination

This clinical trial and the written patient informed consent form have been approved by the medical research ethics committee of the Antoni van Leeuwenhoek on February 24, 2022. Any amendments will be submitted to the ethical committee for approval. The study will be conducted in accordance with the principles of the Declaration of Helsinki and the Medical Research Involving Human Subjects Act (WMO). The protocol has been written and the trial will be conducted according to the ICH Harmonized Tripartite Guidelines for Good Clinical Practice. The study has been registered at ClinicalTrials.gov (NCT05251389).

Documented informed consent will be obtained from all patients before registration in the study in accordance with the national and local regulatory requirements, and conform the ICH guidelines on Good Clinical Practice.

## Discussion

It has long been assumed that the gut microbiome interacts with the immune system, and, more recently, that it contributes to tumor control or escape [4, 5, 11, 23, 27, 28]. Various mechanisms may contribute to such an effect: microbial antigens may stimulate antitumor immune activity through antigenic mimicry, microbes may provide inflammatory or inhibitory signals that influence the activation state of antigen-presenting cells or tumor-specific T lymphocytes, and microbial products may directly or indirectly affect cancer cells [30]. With respect to the role of the microbiome in the antitumor activity of ICI, a growing body of evidence indicates that certain gut microbiota compositions can enhance the function of dendritic cells (DCs) with more potent tumor antigen presentation and cytokine production, increase trafficking of CD4+ memory T cells from intestinal lymph nodes to the TME, decrease Tregs and MDSC numbers, and increase recruitment and activation of interferon-γ-producing tumor-specific effector T cells [4, 5, 11, 18, 30].

Transplantation of fecal microbiota with the aim to restore patients’ perturbed microbiota has accepted and approved for treatment of patients with recurrent *Clostridioides difficile* infection (rCDI). FMT is also studied in various other diseases, such as ulcerative colitis, hepatic encephalopathy, and graft-versus-host disease [24, 26, 35, 36]. However, less is known with regard to the potential role of FMT in the oncological setting. Preclinical and early clinical data showed that transplantation of fecal microbiota can improve anti-tumor immune responses and can reverse the ICI-unresponsive state of a subset of metastatic melanoma patients [4, 5, 11, 23]. However, it is still unclear what the beneficial characteristics of the donor or FMT composition are. One possible explanation is that it is not the composition and origin of the FMT that determine clinical activity, but that the mere alteration of a patient's microbiome can trigger a favorable response. This trial will investigate whether the ICI-response status of FMT donors is a predictor of FMT treatment efficacy.

In the first melanoma FMT trials no serious adverse events were reported [27, 28]. In patients with rCDI, for which FMT is a standard-of-care treatment option, post-FMT adverse events are typically mild and transient, such as diarrhea, cramping, flatulence, belching or constipation. However, rare serious adverse events, including fever, bacteremia, intestinal perforation, aspiration pneumonia, and death, have also been described [37–39]. The risk of infectious complications after FMT, caused by transfer of a multidrug resistant *Escherichia coli* or a shiga-toxin producing *E. coli* through FMT, can be reduced by appropriate donor screening [40]. The donor selection and screening protocols used in this trial, according to international standards, precludes transfer of drug-resistant pathobionts and shiga-toxin producing *E. coli* [32, 41].

The presented double-blinded, randomized phase Ib/II trial will investigate the efficacy and safety of FMT in anti-PD-1 refractory advanced stage melanoma patients, comparing FMTs derived from ICI responding or nonresponding donors. Using feces, blood and tumor samples, we will perform comprehensive analyses looking at the interplay between the gut microbiome, immune cells and clinical response. Outcomes of this study may provide better insights into the optimal FMT composition and background of the FMT-donor, aiming to improve the efficacy of FMT treatment in the oncological setting in future studies.

### Current trial status

The FMT-trial has been open for accrual since April 2022. Currently, donors are being screened for eligibility.

## Supplementary Information

**Additional file 1.**

## Data Availability

FAIR principle and Open Science: The study data will meet the principles of findability, accessibility, interoperability, and reusability [43]. The scientific data will be published in a peer-reviewed open access scientific journal and all collected data and materials will be made accessable for the scientific community. All contributing researchers and organizations will be mentioned in publications and presentations.

## Abbreviations

ALT: Alanine aminotransferase
AST: Aspartate aminotransferase
CD: Cluster of Differentiation
CR: Complete response
CTCAE: Common Terminology Criteria for Adverse Events
DC: Dendritic cell
CTLA-4: Cytotoxic T-lymphocyte-associated protein 4
DNA: Deoxyribonucleic acid
DSMB: Data and Safety Monitoring Board
ECOG: Eastern Cooperative Oncology Group
eCRF: Electronic Case Report Forms
FMT: Fecal microbiota transplantation
ICH: International Council for Harmonisation of Technical Requirements for Pharmaceuticals for Human Use
ICI: Immune checkpoint inhibitors
irAE: Immune-related adverse event
ITS2: Internal transcribed spacer 2
LC–MS/MS: Liquid Chromatography Tandem Mass Spectrometry
LDH: Lactate dehydrogenase
LM: Leptomeningeal
LUMC: Leiden University Medical Center
MDSC: Myeloid-derived suppressor cell
NDFB: Netherlands Donor Feces Bank
NKI: Netherlands Cancer Institute
NMR: Nuclear magnetic resonance
NR: Nonresponding
OS: Overall survival
PCR: Polymerase chain reaction
PD-1: Programmed cell death protein 1
PD-L1: Programmed death-ligand 1
PFS: Progression-free survival
PPI: Proton-pump inhibitor
PR: Partial response
qPCR: Quantative PCR
R: Responding
RECIST: Response evaluation criteria in solid tumors
rCDI: Recurrent *Clostridioides difficile* infection
rRNA: Ribosomal ribonucleic acid
SAE: Serious adverse event
SD: Stable disease
Spp: Species
TME: Tumor microenvironment
Tregs: Regulatory T cells
ULN: Upper limit of normal
WMO: Medical Research Involving Human Subjects Act
