# Supplementary material for: Conversion of unresponsiveness to immune checkpoint inhibition by fecal microbiota transplantation in patients with metastatic melanoma: study protocol for a randomized phase Ib/IIa trial
Source: BMC Cancer. 2022 Dec 30;22:1366. doi: 10.1186/s12885-022-10457-y (PMC9801532; doi:10.1186/s12885-022-10457-y)
Supplement: Supplementary file 1 — Additional file 1. [file 12885_2022_10457_MOESM1_ESM.docx]

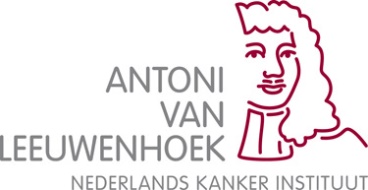
 **Request form – N21FMT**

*Study ID sticker*

Technician:

| Date in freezer | | | | |  |  | Time | | | |  |  |
| --- | --- | --- | --- | --- | --- | --- | --- | --- | --- | --- | --- | --- |
|  | - |  | - |  |  |  |  |  |  |  |  |  |

| **Type of material** | | |
| --- | --- | --- |
| **■**  Stool/Feces |  |  |

| **Additional questions** |
| --- |
| \| **Date** \| \| \| \| \|  \|  \| **Time** \| \| \| \|  \| \| --- \| --- \| --- \| --- \| --- \| --- \| --- \| --- \| --- \| --- \| --- \| --- \| \|  \| - \|  \| - \|  \|  \|  \|  \|  \|  \|  \|  \|  \|   □ On which day did you collect the stool, and at what time?  □ At what time did you put the stool in the fridge?     \| **Time** \| \| \| \|  \| \| --- \| --- \| --- \| --- \| --- \| \|  \|  \|  \|  \|  \|  \|   □ What did the stool look like?  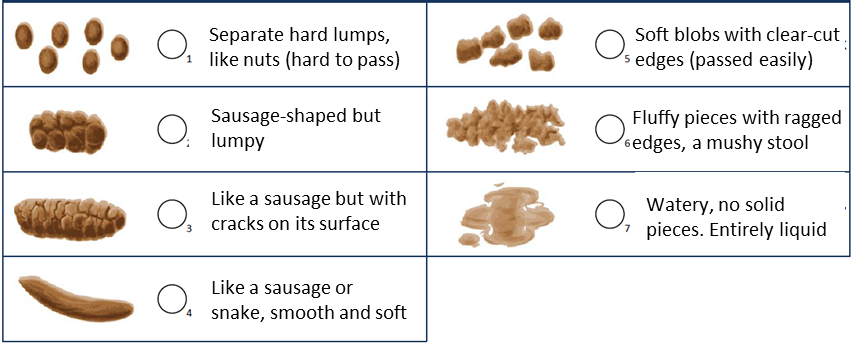  □ Have you used any nutritional supplements (e.g. vitamins, probiotics*) in the past three months? If yes, which one? *A probiotic is a food supplement that affects the intestinal flora, such as Yakult, Activia, Vifit, Actimel.  ………………………………………………………………………………………………………………………………………………………………………  ………………………………………………………………………………………………………………………………………………………………………  □ Do you follow a special diet (e.g. gluten-free, vegetarian, vegan etc). If yes, what kind of diet?  ……………………………………………………………………………………………………………………………………………………………………….  ……………………………………………………………………………………………………………………………………………………………………….  □ How many times a week do you have stools? ……………………………………………………………………………………………………………………………………………………………………….  ………………………………………………………………………………………………………………………………………………………………………. |
|  |
